# Integrating Exercise and Education into Lung Cancer Care: Results from the OVER-CRF Pilot Study on Cancer-Related Fatigue and Quality of Life

**DOI:** 10.3390/curroncol33060313

**Published:** 2026-05-27

**Authors:** Maria Beatrice Galavotti, Alessia Pecorari, Carlotta Mainini, Monica Denti, Monica Messori, Stefania Costi, Barbara Bressi, Martina Pellegrini, Patrizia Ciammella, Francesco Falco, Francesca Zanelli, Luca Braglia, Stefania Fugazzaro

**Affiliations:** 1 Physical Medicine and Rehabilitation Unit, Azienda USL-IRCCS di Reggio Emilia, 42123 Reggio Emilia, Italymonica.denti@ausl.re.it (M.D.);; 2 Research, EBP & IRCCS Unit, Health Professions Department, Azienda USL-IRCCS di Reggio Emilia, 42123 Reggio Emilia, Italy; 3 Department of Surgery, Medicine, Dentistry and Morphological Sciences, University of Modena and Reggio Emilia, 41125 Modena, Italy; 4 Radiation Oncology Unit, Azienda USL-IRCCS di Reggio Emilia, 42123 Reggio Emilia, Italy; 5 Pneumology Unit, Azienda USL-IRCCS di Reggio Emilia, 42123 Reggio Emilia, Italy; 6 Medical Oncology, Azienda USL-IRCCS di Reggio Emilia, 42123 Reggio Emilia, Italy; 7 Clinical Trial Center–Statistics Unit, SOC Infrastructure, Research and Statistics, Azienda USL-IRCCS di Reggio Emilia, 42123 Reggio Emilia, Italy

**Keywords:** rehabilitation, lung neoplasm, chemotherapy, radiotherapy, fatigue, pulmonary rehabilitation, education, self-management, feasibility

## Abstract

**Background:** Cancer-Related Fatigue (CRF) significantly impairs physical performance and quality of life (QoL) in patients with non-small-cell lung cancer (NSCLC). The OVER-CRF study evaluated the feasibility, safety, and preliminary efficacy of a personalized pulmonary rehabilitation (PR) program combining supervised exercise and education during active treatment. **Methods:** Patients with stage II–III NSCLC were randomized to Early-PR (initiated at the start of anticancer therapy) or Delayed-PR (initiated three months later). The 3-month intervention included two educational sessions and eight supervised exercise sessions. The primary outcome was adherence; secondary outcomes included safety, CRF (FACIT-FS), QoL (EORTC-QLQ-C30), and physical performance (6MWT). **Results:** Thirty-one patients were randomized (mean age 67.4 years). Adherence was excellent (Early: 86.7%; Delayed: 91.7%), exceeding feasibility thresholds. No exercise-related adverse events occurred. At 12 months, 50% of participants showed clinically meaningful CRF improvements. While both groups improved 6MWT performance during the intervention, the Delayed-PR group demonstrated more sustained QoL improvements from T1 through T3 compared to the Early-PR group. The dropout rate (25.8%) was consistent with the existing literature. **Conclusions:** Personalized PR is feasible and safe for NSCLC patients undergoing multimodal therapy. While early intervention provides immediate benefits, initiation timing may influence long-term QoL trajectories. These findings support integrating exercise and education into standard oncological care pathways.

## Simple Summary

Patients with lung cancer often suffer from severe exhaustion, known as cancer-related fatigue, which affects their quality of life and physical performance. The study evaluated a 3-month program combining supervised exercise and education on symptom management during anticancer treatment. Two groups were compared: one initiating the program immediately at the start of anticancer therapy and another starting three months later. Results showed that this approach is feasible and safe, as demonstrated by high adherence rates and the absence of adverse events. While health outcomes improved in both groups during the intervention, more sustained improvements in quality of life were observed at the long-term follow-up in the group that started the program later. These findings suggest that exercise and education should be integrated into cancer care to help patients remain active and improve well-being during and after oncological treatment.

## 1. Introduction

Lung cancer remains one of the most frequently diagnosed malignancies globally, with approximately 2.5 million new cases recorded in 2022 [1]. In Europe, it accounts for an estimated 11% of all cancer diagnoses, corresponding to 314,857 new cases in 2024 [2]. Patients often experience a substantial burden of disabling symptoms—most notably dyspnea and cancer-related fatigue (CRF)—which severely impair physical performance and health-related quality of life (QoL) [3,4].

CRF is defined as a persistent, subjective sense of physical, emotional, or cognitive exhaustion that is disproportionate to recent activity. It often begins at diagnosis and may persist for years after the completion of treatment, creating a major barrier to social reintegration [5]. Indeed, CRF is one of the most prevalent and debilitating symptoms experienced by individuals with lung cancer, affecting up to 80% of survivors [5,6].

Current clinical guidelines, including those from the European Society for Medical Oncology (ESMO) and the National Comprehensive Cancer Network (NCCN), advocate for non-pharmacological interventions as the gold standard for CRF management [5,7]. Specifically, pulmonary rehabilitation (PR) programs—including moderate-intensity aerobic exercise, muscle strengthening, and therapeutic education—have demonstrated effectiveness in improving lung function, exercise tolerance, and psychological well-being in patients with respiratory diseases, including lung cancer [8,9,10,11,12]. Moreover, evidence suggests that these interventions can counteract the side effects of antitumor therapies, such as muscle mass loss and treatment-induced deconditioning, without significant adverse effects [9,12,13,14]. Recent observational evidence further supports the association between higher physical activity levels, better physical fitness, and improved quality of life in patients with lung cancer undergoing chemotherapy [15].

Therapeutic education is also an integral component of a successful PR program in patients with lung cancer. It is recommended to empower patients and caregivers through a deep understanding of the multifactorial nature of cancer-related fatigue, fostering self-management skills through activity pacing and coping strategies, and helping individuals identify personal fatigue patterns to improve their overall QoL [7]. The benefits of exercise are well known, with research highlighting its role in reducing mortality in cancer patients [16]. Cardiorespiratory fitness is a key independent predictor of survival; higher levels correlate with lower all-cause and cancer-specific mortality, including in lung cancer patients [17,18]. Implementing a tailored exercise prescription to improve muscle strength and cardiorespiratory fitness may play a crucial role in reducing cancer-related mortality in this population [18].

Despite this, the optimal timing for integrating PR into the lung cancer care continuum remains a critical area of uncertainty. While preliminary data support the safety and efficacy of exercise both during and after treatment [19,20,21,22], many patients remain sedentary due to treatment-related toxicities or environmental barriers [20,23,24]. It is therefore unclear whether an “early” intervention, initiated at the onset of non-surgical therapies (chemotherapy, radiotherapy, immunotherapy, etc.), offers better feasibility, tolerability, and clinical outcomes than a “delayed” approach implemented later in the care pathway.

The aim of this study was to explore the timing of rehabilitation intervention for patients undergoing non-surgical cancer treatments and to determine the most feasible timing in terms of tolerability and adherence to the protocol.

Secondary objectives included assessing recruitment rates, safety, and the program’s impact on CRF, physical performance, and overall well-being.

## 2. Materials and Methods

### 2.1. Study Design

This was a drug-free interventional study, a two-arm parallel, single-center, randomized controlled trial (RCT). This study protocol complied with the 2010 CONSORT statement [25]. Protocol details were registered in www.clinicaltrials.gov (NCT06051136) and have been extensively described in a previous publication [26].

The OVER-CRF (Overcome Cancer-Related Fatigue) was a pilot study designed to investigate the feasibility of a pulmonary rehabilitation program consisting of supervised exercise and educational sessions for patients with stage II or III non-small-cell lung cancer (NSCLC) undergoing active treatment. The primary objective was to evaluate patients’ adherence to the program when rehabilitation was offered early (at the initiation of anticancer treatment) versus when it was delayed by three months. Study participants were randomized into two groups: Early PR (immediate start) and Delayed PR (start after three months).

The primary objective was to assess patient adherence to the PR program when initiated either at the start of non-surgical lung cancer treatment (Early) or three months later (Delayed). The Delayed PR group served as the control arm. Secondary objectives included evaluating:

- recruitment rates, treatment failure rates (overall and by cause), 12-month survival, and intervention safety;
- clinical outcomes, including CRF, QoL, physical performance, and physical activity levels during the 12 months following enrollment.

### 2.2. Study Setting and Recruitment

#### 2.2.1. Study Setting

OVER-CRF was a pilot RCT conducted in the Physical Medicine and Rehabilitation Unit within the cancer care network at the Santa Maria Nuova Hospital of Reggio Emilia (Italy), a Comprehensive Cancer Center accredited by the European Organization of Cancer Institutes. The Physical Medicine and Rehabilitation Unit provides clinical rehabilitation for approximately 80–90 lung cancer patients per year.

#### 2.2.2. Participants and Recruitment Process

Patient screening and enrollment occurred between 28 February 2023 and 31 December 2024.

Inclusion criteria: patients with a new diagnosis of stage II or III NSCLC who were candidates for non-surgical cancer therapies (chemotherapy, radiotherapy, and/or immunotherapy), with a prognosis of ≥12 months.

Exclusion criteria: patients with barriers to participation in the PR program (e.g., cognitive impairment, severe pre-existing disability, or psychiatric disease), language barriers preventing comprehension of the physiotherapist’s rehabilitation instructions, and patients scheduled for lung surgery as a single treatment.

Patients undergoing multimodal therapy (lung surgery plus adjuvant/neoadjuvant treatment) were eligible and could be enrolled either at the start of non-surgical cancer therapies or at least one month post-surgery.

Multidisciplinary healthcare professionals (oncologists, radiotherapists, pulmonologists, physiatrists, physiotherapists, etc.) screened patients for eligibility. Upon confirmation, the physiatrist provided detailed study information and obtained written informed consent. For patients undergoing surgery, enrollment was postponed one month after surgery to prevent evaluation bias caused by a temporary decline in functional performance and post-operative pain.

Any refusal to participate in the study was recorded, along with its reason, when shared spontaneously.

#### 2.2.3. Measurements and Data Collection Timepoints

The baseline (T0) assessment was conducted at the beginning of antitumor treatments, specifically within 30 days of the non-surgical treatment recommendation (chemotherapy, radiotherapy, and/or immunotherapy). Baseline information was collected for each participant, including sociodemographic (age, sex, family composition, education level, occupation, and smoking habits) and clinical information (performance status, cancer stage, and other clinical data). A detailed description of the measurement tools used in the trial is provided in the report of the study protocol [26].

**Figure 1:**
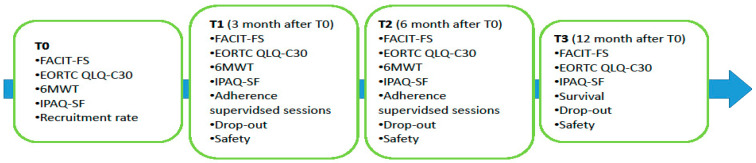
The assessment timeline.

Figure 1 shows the assessment timeline.

### 2.3. Intervention

The PR intervention lasted 3 months and consisted of eight individual supervised exercise sessions and two group therapeutic education sessions; the types of exercises and their intensity were defined and delivered according to specific exercise guidelines for cancer [9,27]. The supervised PR sessions were scheduled every 7–14 days, led by a physiotherapist specialized in pulmonary and cancer rehabilitation. Each supervised PR session included aerobic, muscle-strengthening, balance, flexibility, and respiratory training (see Table 1), tailored to the patients’ characteristics. Moderate-intensity aerobic exercise was proposed two to three times per week, and muscle strength training two times per week, according to guideline recommendations for cancer survivors [27]. Patients were encouraged to perform moderate-intensity aerobic exercise based on their health status from week to week (minimum twice a week, suggested three times). Respiratory exercises were performed every day. Exercise intensity was progressively increased over time based on patient adherence and performance by adjusting duration, weight, number of sets, or repetitions. Both patients and caregivers were offered two educational sessions on CRF management and active lifestyles (Table 1), supplemented by a booklet summarizing the core educational content.

The in-person exercise and educational sessions lasted approximately 60 min. Following the initial in-person training, subsequent 30 min sessions were available via telerehabilitation upon patient request. To promote adherence, PR sessions were scheduled flexibly, according to the patients’ needs and the timing of hospital appointments for visits or therapies.

Throughout the trial, patients received all therapies and clinical follow-up as part of their usual care. In cases of worsening of clinical conditions or adverse events, the PR program was modified or suspended after consultation with a physician (physiatrist, oncologist, or pulmonologist).

### 2.4. Outcomes

The primary outcome was adherence. We considered a patient “adherent” if they attended at least 70% of the supervised PR sessions scheduled for the relevant allocation group [19].

Secondary outcomes included:

- Recruitment rates, treatment failures and reasons for drop-out, 12-month survival rates;
- Safety: adverse events related to the PR program during the 3-month intervention were recorded (falls, fractures, muscle pain, exertional dyspnea, acute musculoskeletal inflammation, hematomas, etc.); falls or fractures occurring between T2 and T3 were also monitored;
- Clinical assessments: CRF was evaluated using the Functional Assessment of Chronic Illness-Fatigue Scale (FACIT-FS), QoL with the European Organization for Research and Treatment of Cancer Quality of Life Questionnaire-C30 (EORTC-QLQ-C30), and physical performance with the 6-Minute Walk Test (6MWT);
- Physical activity levels were assessed using the International Physical Activity Questionnaire–Short Form (IPAQ-SF).

A detailed description of the measurement tools used in the trial is included in the study protocol report [26].

Figure 1 shows the assessment timeline.

### 2.5. Sample Size

There is no literature available on the feasibility of a combined intervention (physical exercise and therapeutic education) in lung cancer patients during adjuvant therapies. As a pilot study, a formal sample size calculation was not performed, according to the CONSORT 2010 statement: extension to randomized pilot and feasibility trials [25]. The research group aimed to enroll 40 participants to provide sufficient feasibility data.

### 2.6. Allocation and Blinding

Participants were allocated 1:1 using block randomization. Randomization was conducted online, and allocation assignments were based on a previously generated block randomization list with a block size of four.

While blinding the treating physiotherapist and patients was not possible, a single-blind design was maintained; independent researchers conducting follow-up assessments and data analysis were blinded to group assignments.

### 2.7. Data Analysis

Data were analyzed using the R statistical software version 4.5.0. Given the small sample size, for each calculated percentage, the exact two-sided confidence interval was calculated according to the Clopper–Pearson approach, ensuring a confidence level of at least 95%.

The statistical analysis of the variables included among the secondary outcomes was descriptive, with tabular presentation of means, standard deviations, and the most appropriate measures of central tendency (with 95% CI), selected on the basis of the distribution characteristics of the variables under study.

Adherence was calculated as the ratio between the number of sessions completed and the number of sessions scheduled. Participants were deemed “adherent” to the protocol if they completed ≥70% of the planned sessions.

Improvements in CRF, QoL, and physical performance were evaluated by comparing changes between groups in FACIT-FS, EORTC-QLQ-C30, and 6MWD scores exceeding their MCID. The percentages of patients in both groups that exceeded the MCID for FACIT-FS, EORTC-QLQ-C30, and 6MWD were also calculated (MCID: 3 points [28], 5 points [29], and 42 m [30], respectively).

In cases of dropout, the date and reason for withdrawal from the protocol were recorded when spontaneously provided (withdrawal of informed consent, worsening of clinical conditions, death, lack of motivation to participate in the study, unavailability, lost to follow-up, or other).

No missingness was expected for the primary outcome; statistical analyses for secondary outcomes were performed on a complete-case basis.

### 2.8. Patient and Public Involvement

No patients or associations were involved in the design or conduct of the study. However, the development of the rehabilitation program was based on a thorough literature review, and patient priorities were collected by specialists during routine clinical practice.

## 3. Results

### 3.1. Recruitment and Participant Flow

Of the 67 eligible individuals identified, 31 patients were enrolled in the study. Fifteen patients were allocated to the experimental group (Early PR) and sixteen to the control group (Delayed PR).

**Figure 2:**
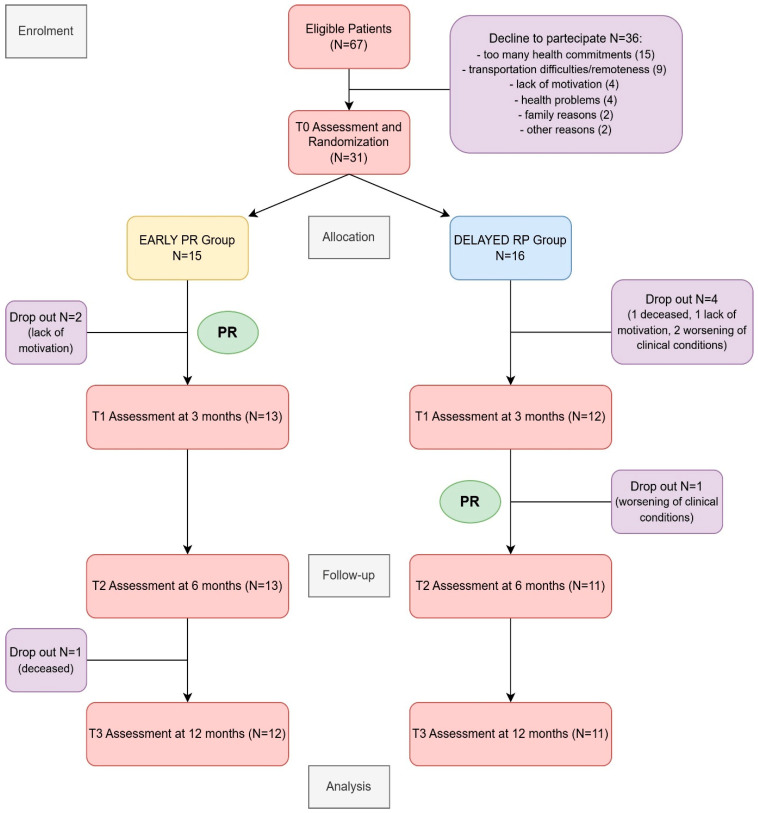
Flowchart of the OVER-CRF project.

The overall recruitment rate was 46.3%. Thirty-six patients (53.7%) declined to participate, primarily due to the high frequency of appointments related to their planned oncological treatments or transport-related barriers. Figure 2 presents the OVER-CRF study flowchart.

### 3.2. Baseline Characteristics of the Sample

Baseline demographic and clinical characteristics are summarized in Table 2.

The cohort predominantly consisted of older adults (mean age 67.4 years, Early PR group 66.6 years, Delayed PR group 68.1 years); 48% of the total sample was over 70 years old (Early PR group 40%; Delayed PR group 56%). Sex distribution was balanced between the two groups. Most participants lived with their families (81%), and approximately two-thirds were retired. Regarding cancer stage, 19% of participants had stage II and 81% had stage III NSCLC, with a similar distribution across both groups. At the time of enrollment, 39% of participants were current smokers.

### 3.3. Anticancer Treatments

Table 2 also outlines the antineoplastic treatments administered during the PR intervention.

Most patients (90%) received outpatient intravenous chemotherapy or immunotherapy. Sixty-one percent underwent radiotherapy: 32% received concurrent radiotherapy (five times per week for 5–6 weeks), also administered on an outpatient basis, and 29% received sequential radiotherapy (five times per week for 5–6 weeks), following the completion of their chemo/immunotherapy schedule.

One patient underwent radiotherapy alone, and two patients were treated with osimertinib, an oral tyrosine kinase inhibitor (TKI).

### 3.4. Adherence and Safety of the Rehabilitation Intervention

The mean duration of supervised exercise sessions was 58.4 min (SD 5.1), while therapeutic education sessions had a mean duration of 55 min (SD 2.7).

Exercise sessions delivered via telerehabilitation accounted for only 2.4% of the total sessions: this involved one patient in the Early PR group (4 sessions) and one patient in the Delayed PR group (1 session). The mean duration of telerehabilitation sessions was 29.4 min (SD 3.1).

Regarding the primary outcome, 13 of the 15 patients (86.7%) in the Early PR group were adherent, attending at least 70% of the planned sessions. In this group, one patient attended 50% of the sessions before withdrawing, and another withdrew prior to any session. In the Delayed PR group, four patients withdrew before the intervention began, leaving 12 patients to start rehabilitation three months post-enrollment (see Table 3). Eleven out of those 12 patients (91.7%) achieved the 70% adherence threshold, while one patient attended 50% of the sessions because of worsening clinical conditions. In summary, of the 31 study participants, excluding the 7 dropouts, all 24 remaining participants were adherent to the treatment program.

The overall dropout rate was 25.8% (*n* = 8): 3 patients in the Early PR group and 5 in the Delayed PR group. Reasons for dropout included worsening clinical conditions (*n* = 3), lack of motivation (*n* = 3), and death (*n* = 2).

No adverse events related to exercise were recorded, and no falls or fractures occurred during the T1–T2 period.

The 12-month survival rate was 93.3% in the Early PR group (one death occurred due to respiratory failure between T2 and T3) and 93.8% in the Delayed PR group (one death due to SARS-CoV-2 infection occurred between T0 and T1).

### 3.5. Clinical Outcomes

**Figure 3:**
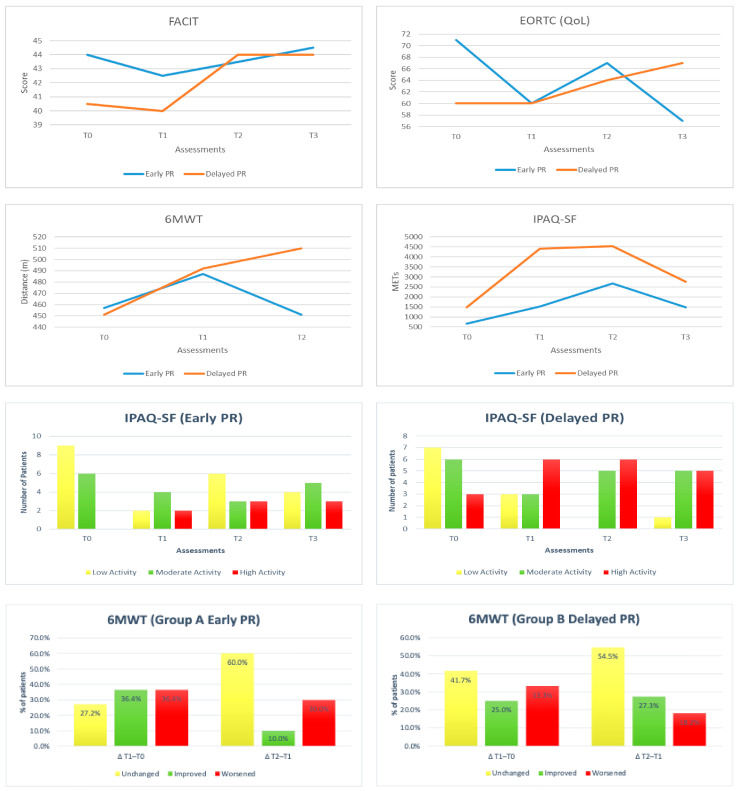
Changes in secondary outcomes over time.

Changes in secondary clinical outcomes are detailed in Table 4 and in Figure 3.

Table 4 presents data for all participants enrolled in the study; Figure 3 graphically illustrates data for participants who adhered to the rehabilitation program (Early-PR *N* = 13; Delayed-PR *N* = 11). More specifically, for the Early-PR group, analyses included 11 participants for the T1–T0 interval, 10 participants for the T2–T1 interval, and 12 participants for the T3–T0 interval. Regarding the Delayed-PR group, 12 participants were analyzed for the T1–T0 interval, while 11 participants were included in both the T2–T1 and T3–T0 intervals.

- **Cancer-Related Fatigue (FACIT-FS)**: Perceived fatigue improved in both the Early and Delayed groups following the PR intervention (+2.9 vs. +3.9, respectively).

In the Early PR group, fatigue improved during the first three months, fluctuated at six months, and improved again by the one-year mark. The Delayed group remained stable for the first three months, showed significant improvement at six months (post-PR), and experienced a slight decline at 12 months.

Considering the FACIT MCID of 3 points reported by Cella [28], at 3 months, 29% of patients had clinically significant improvement in fatigue (57% in the Early PR group, immediately after PR, and 43% in the Delayed PR group, before PR). Six months after enrollment (T2), 30% of patients showed a clinical improvement in fatigue, precisely 43% in the Early PR group and 57% in the Delayed PR group, immediately after rehabilitation. At T3 (12 months after enrollment), 43% of patients showed a clinical improvement in fatigue (50% in both groups).

- **Quality of Life (EORTC QLQ-C30):** The groups showed diverging trends. The Early group’s QoL worsened initially (T0–T1), improved at six months, and declined again at 12 months. Conversely, the Delayed group remained stable initially and subsequently showed progressive and sustained QoL improvements from T1 through T3.

Considering the EORTC subdomain scores, the EORTC functional score in the Early PR group remained the same between T0 and T1 but declined at T2, with a subsequent improvement at T3, and this pattern was consistent with the 6MWT results.

Regarding symptoms, the EORTC symptom score declined between T0 and the subsequent assessment, suggesting a lighter symptom burden. In the Delayed PR group, the perceived symptoms improved between T0 and T1, remained stable at 6 months, but benefits were not maintained over time.

- **Physical Performance (6MWT):** Mean walking distance improved in both groups (Early: +30.2 m; Delayed: +17.6 m). Although mean changes did not reach the 42 m MCID threshold described by Granger et al. for lung cancer patients [30], a subset of the sample achieved this level of improvement (36% in the Early group and 25% in the Delayed group).
- **Physical Activity (IPAQ-SF):** Baseline activity levels were low, with 100% of the Early group and 81% of the Delayed group categorized as inactive. Post-intervention assessments showed a decrease in inactive patients in both groups, suggesting the PR program effectively increased routine physical activity levels. This active lifestyle was largely maintained between T2 and T3.

## 4. Discussion

The OVER-CRF study aimed to evaluate the feasibility, safety, and effects of a personalized pulmonary rehabilitation program for stage II–III NSCLC patients. The intervention combined supervised exercise and education delivered during active treatment, either at the start of anticancer therapy (Early PR) or three months later (Delayed PR). The OVER-CRF pilot study was planned according to real-life clinical pathways for lung cancer patients, and its results provide useful data to explore hypotheses with good translational potential in future research.

The recruitment rate of 46.3% is slightly higher than rates reported in similar lung cancer rehabilitation studies [31,32]. However, the fact that 53.7% of eligible patients declined to participate is noteworthy. Given that PR has been shown to be a safe intervention for this population, this high refusal rate may reflect limited awareness among specialists regarding the benefits of PR. Consideration should also be given to patients’ own difficulties in engaging with rehabilitation programs. Although the evidence on the reasons why cancer patients decline rehabilitation remains limited, physical, psychosocial, and practical barriers have been described [33]. Future studies should explore how these individual and structural factors intersect, with a specific focus on frail populations, to better understand how to enhance access to care and participation in research studies.

Considering sample baseline characteristics, our study further confirmed the feasibility of PR in patients with advanced disease—most of whom were undergoing active treatment—irrespective of whether PR was delivered early or in a delayed setting. Rather than being viewed as an optional or ancillary service, PR should be systematically integrated into standard care pathways for lung cancer patients.

Our primary outcome revealed excellent adherence rates in both the early (86.7%) and delayed (91.7%) intervention groups. These figures exceed the 70% threshold typically used to define feasibility in exercise oncology. This high adherence may be attributed to the research team’s scheduling flexibility; by synchronizing PR sessions with oncology appointments, the travel burden on participants was reduced. This approach likely met patient needs, enhanced motivation, and improved session attendance. Moreover, the booklet provided to patients at the beginning of the pulmonary rehabilitation program, designed to promote patient empowerment during cancer care, in combination with group educational sessions, may have facilitated increased physical activity levels and promoted positive changes in exercise habits, as previously reported in the literature [34].

Interestingly, despite the availability of telerehabilitation, only 2.4% of sessions were conducted remotely. This strong preference for in-person attendance, particularly among the older cohort (mean age 67.4), aligns with findings by Avancini et al. [35], who also reported that 72% of Northern Italian cancer patients preferred receiving exercise instructions face-to-face. This suggests that Italian cancer patients highly value the interpersonal aspect of supervised care and the peer support fostered by group settings.

Nevertheless, as digital literacy grows, telemedicine may become a more widely accepted tool to facilitate recruitment and adherence in the coming years.

The dropout rate was higher in the Delayed PR group compared to the Early PR group (31% vs. 20%). Most attrition in the delayed group occurred while patients were waiting to begin the intervention, suggesting these dropouts were unrelated to the PR program itself, but this hypothesis should be explored in future studies with larger samples. The primary reasons for attrition are consistent with previous cancer rehabilitation trials. High drop-out rates have been reported in similar RCTs involving lung cancer populations [31,32].

Crucially, the program was safe, with no exercise-related adverse events reported during rehabilitation. This is in line with other studies [36] and reinforces the evidence that even intensive multimodal therapies (e.g., concurrent chemoradiotherapy) are not contraindications for supervised physical activity. Safety standards for exercise in cancer patients have been summarized in guidelines from the ACSM [37]. Our expert physiotherapists tailored the rehabilitation program for each patient, and patients were supervised while performing the exercises throughout the 3-month intervention period; week by week, individualized exercise prescriptions were dynamically adapted to the clinical condition of each participant. Tailoring exercise programs for each patient is a key element for safety and for patient adherence. Recent randomized evidence further supports the role of personalized exercise prescription in improving fatigue and physical function outcomes in lung cancer patients [38].

Secondary objectives included assessing changes in CRF, physical performance, overall well-being, and physical activity levels in the first 12 months after enrollment: the descriptive analyses of our pilot study support exploratory considerations.

Our findings confirm the beneficial role of exercise in managing CRF, with up to 57% of the sample achieving a clinically meaningful reduction in fatigue. These results align with the high-impact meta-analyses by Mustian et al. [39], which identify exercise as superior to pharmaceutical interventions for CRF. In this context, recent findings by Van Hulle et al. [6] further emphasize how the trajectory of fatigue is closely related to the type of treatment and level of physical activity, suggesting that early and personalized exercise interventions are crucial to mitigate symptom worsening. Exercise may counteract the biological drivers of fatigue—declines in cardiorespiratory fitness, muscle wasting, and inflammation [36]—and positive effects have specifically been observed in patients undergoing chemotherapy [15] or radiotherapy [40]. However, further studies are required to explore the true effect size and to establish how exercise prescriptions should be personalized based on individual patients’ characteristics.

In our study, the combination of physical activity and therapeutic education likely empowered patients to better manage their energy levels through “activity pacing”.

A key observation was the difference in QoL trajectories. While the Early PR group experienced fluctuations, the Delayed PR group showed a more sustained and progressive improvement in QoL from T1 through T3. This suggests that rehabilitation initiated slightly later (3 months after the start of anticancer treatment) might coincide with a period of greater clinical stability. Another key element could have been patient psychological “readiness” for rehabilitation 3 months after beginning oncological treatments: this was a clear clinical impression among the physiotherapists supervising participants, but it should be verified with specific assessments in future studies, to investigate the “optimal window” for rehabilitation within the lung cancer care pathway.

Good evidence indicates that structured patient education and care interventions prevent decline in QoL for cancer patients undergoing radiotherapy, supporting cancer survivors’ emotional and social well-being, empowering patients to self-manage symptoms and engaging them in strategies to cope with cancer and the treatment journey [41].

While higher physical activity levels correlate positively with global health status and improved QoL in lung cancer patients [15], research findings remain inconsistent. Despite the promise of exercise as a strategy, its reported effects on QoL continue to be highly variable [20,21,22,37,42].

Both groups showed improvements in physical performance (6MWT) during their respective intervention periods. These findings are consistent with recent meta-analytic evidence confirming the beneficial effects of exercise interventions on physical function in lung cancer patients [42]. In the Early PR group, 36% of patients exceeded the Minimal Clinically Important Difference (MCID) of 42 m immediately after the program. However, the slight decline observed at 6 months (T2) suggests a “detraining” effect once supervision ends. This pattern highlights the necessity of transitioning from supervised programs to long-term maintenance strategies to preserve functional gains during the continuum of cancer care.

Encouragingly, data from the IPAQ-SF indicated a successful shift from sedentary to active lifestyles. The reduction in inactive patients was maintained even at the 12-month follow-up, suggesting that the program facilitated sustainable behavioral changes.

In summary, the data support the positive effect of personalized physical rehabilitation programs in patients with lung cancer, highlighting immediate and medium-term benefits in terms of functional capacity, adoption of active behaviors, reduction in fatigue, and improvement in quality of life. However, our findings warrant further investigation into the “optimal timing” for rehabilitation along the lung cancer care journey.

Given increased treatment options and gradually improving survival, a future challenge is the integration of exercise into lung cancer care, enhancing collaboration among healthcare professionals in cancer rehabilitation referral pathways. This need is consistent with emerging evidence supporting structured and longitudinal supportive care models for lung cancer patients, aimed at improving overall care pathways and patient outcomes [43].

Researchers should explore how exercise can complement other treatments and interventions delivered as adjunctive therapies by qualified professionals—such as clinical exercise physiologists and physiotherapists—as part of the care team. Addressing the needs of a heterogeneous patient population, including older adults and other subgroups, is an important consideration for improving adherence in lung cancer patients: a goal-setting approach, expert clinicians, and the use of technological devices may facilitate patient recruitment and maintenance of an active lifestyle.

### Limitations and Strengths

The primary limitation is the small sample size and the dropout rate (25.8%), which was largely driven by disease progression rather than the intervention itself. As a pilot study, these results are not intended for broad generalization but provide a robust foundation for future trials.

Another potential limitation of this study is the relatively low volume of supervised exercise (8 sessions over 3 months), which is below the intensity typically recommended by international guidelines [37]. This reduced dosage might have limited the magnitude of overall physical improvements. However, this was a pilot study designed to maximize feasibility and adherence in a population undergoing intensive multimodal therapy, where the burden of frequent hospital-based clinical appointments is a known barrier [24]. Notably, the integration of a comprehensive educational component focused on self-management (including goal setting, problem-solving, and breathing techniques) was designed to provide additional support for patients throughout the treatment pathway. This combined approach may have contributed to the sustained improvements in QoL observed at the long-term follow-up.

The study’s strengths include its randomized design, a structured educational approach and tailored rehabilitation intervention, a long-term 12-month follow-up, and the use of validated MCID values to ensure clinical relevance.

## 5. Conclusions

Personalized pulmonary rehabilitation in lung cancer patients is feasible and safe, whether initiated at the start of systemic anticancer treatment or three months later. The OVER-CRF program effectively improved functional capacity, perceived fatigue, and adoption of active behaviors. Direct clinician contact, group sessions, and organizational flexibility were central to supporting adherence, though the timing of the intervention may uniquely influence long-term QoL. Further large-scale efficacy studies are required to define the optimal protocols for improving quality of life, physical performance, and management of cancer-related fatigue in lung cancer patients.

## Figures and Tables

**Table 1:** Overview of the OVER-CRF PR intervention.

Individual PR program offered to patients during physiotherapist-supervised sessions	Main themes addressed in therapeutic educational group sessions
**Type of exercise (FITT-VP, tools and monitoring)**	**First group session:** Empowering people with evidence-based strategies to enhance physical well-being and overall QoL.
**Aerobic exercise** 20–30 min, 2–3 times per week Bicycle Intensity: 60–80% of maximum heart rate Increase by 5 min every 4 weeks. **Tools and monitoring:** Oximeter, heart rate, Borg Scale, resistance applied	**Goal Setting:** helping participants set realistic short- and long-term goals. **Problem Solving:** identifying potential barriers and solutions and evaluating results. **Physical Activity:** brainstorming sessions on the benefits of physical exercise. **Assistive Devices and Orthoses:** Guidance on the use of walking aids and supportive devices to enhance mobility and reduce disability. **Breathing Techniques and Relaxation:** diaphragmatic breathing and body-scan relaxation exercises to manage stress and fatigue and promote well-being.
**Muscle strengthening** 20–30 min, 2 times per week Isometric and isotonic exercises for the lower limbs, upper limbs, and trunk Repetitions: 8–15; Sets: 2–4 Gym machines, ankle weights, exercise bands, balls/cushions **Tools and monitoring:** Weight, number of repetitions/sets, speed, breathing, posture, and Borg Scale	**Second session:** Empowering people to self-manage cancer-related fatigue.
	**Goal Setting:** training people to set realistic short- and long-term goals. **Problem Solving:** identifying potential barriers and solutions and evaluating results. Helping patients plan strategies to incorporate physical activity into everyday life. **Understanding Fatigue:** exploring the causes and symptoms of CRF through brainstorming sessions to share participants’ experiences and discuss strategies to manage fatigue. **Effective Communication:** empowering patients to ask for help and express their needs to caregivers and healthcare professionals, using role-playing techniques to improve communication skills. **Relaxation Techniques:** diaphragmatic breathing and body-scan relaxation exercises to manage stress and fatigue and promote well-being. **Self-management of breathing exercises:** empowering people through the use of specific breathing exercises, including PEP therapy and incentive spirometer, to improve respiratory function and alleviate fatigue-related symptoms.

**Table 2:** Baseline demographic and clinical characteristics of participants and anticancer treatments offered during the OVER-CRF project.

Variables		Overall (*N* = 31)	Early PR (*N* = 15)	Delayed PR (*N* = 16)
**Sex**	Female (*N*, %)	12 (39)	6 (50)	6 (50)
	Male (*N*, %)	19 (61)	9 (47)	10 (53)
**Age, years (mean, SD)**		67.4 (9.36)	66.6 (11.5)	68.1 (7.14)
**Age ≥ 70 years (*N*, %)**		15 (48)	6 (40)	9 (56)
**Education, years (mean, SD)**		10.2 (3)	10.5 (3.31)	9.94 (2.74)
**Family status**	Living with family (*N*, %)	25 (81)	12 (80)	13 (81.25)
	Living alone (*N*, %)	5 (16)	3 (20)	2 (12.5)
	Living with friends (*N*, %)	1 (3)	0 (0)	1 (6.25)
**Occupation**	Retired (*N*, %)	21 (68)	11 (73.4)	10 (62.5)
	Employed (*N*, %)	8 (25)	2 (13.3)	6 (37.5)
	Unemployed (*N*, %)	2 (7)	2 (13.3)	0 (0)
**Lung cancer stage**	II	6 (19)	3 (20)	3 (18.75)
	III	25 (81)	12 (80)	13 (81.25)
**Smoking habit**	Current smoker	12 (39)	9 (60)	3 (18.75)
	Former smoker (quit ≥ 6 months)	16 (51)	5 (33)	11 (68.75)
	Never smoker	3 (10)	1 (7)	2 (12.5)
**Performance status**	ECOG 0 (*N*, %)	22 (71)	11 (50)	11 (50)
	ECOG 1 (*N*, %)	9 (29)	4 (44)	5 (56)
**Anticancer treatment** **during PR**	**IV Chemotherapy** (Carbo/Cisplatin + gemcitabine; Cisplatin + etoposide; Carbo/Cisplatin + Paclitaxel; Carboplatin + Taxol NEOADJ; Cisplatin + Vinorelbin)	10 concurrent RT 8 sequential RT 7 no RT	6 concurrent RT 4 sequential RT 2 no RT	4 concurrent RT 2 sequential RT 5 no RT
	**IV Chemotherapy + Immunotherapy** (Carboplatin + Pemetrexed + Pembrolizumab; NEOADJ Carboplatin + Pemetrexed + Nivolumab)	2 sequential RT 3 no RT	- 2 no RT	2 sequential RT 1 no RT
	**No IV chemotherapy/immunotherapy**	1 only RT	1 only RT	-
	**Oral TKI (Osimertinib)**	1 sequential RT 1 no RT	- -	1 sequential RT 1 no RT

**Table 3:** Feasibility and safety of the OVER-CRF rehabilitation program.

Rehabilitation Intervention	Overall (*N* = 31)	Early PR (*N* = 15)	Delayed PR (*N* = 16)
**Started the PR program according to allocation (*N*, %)**	27 (87)	15 (100)	12 (75)
**Attended >70% of sessions according to allocation (*N*, %)**	24 (77)	13 (86.7)	11 (91.7)
**Drop-out (*N*, %)**	8 (26)	3 (20)	5 (31)
**Exercise-related adverse events (*N*, %)**	0 (0)	0 (0)	0 (0)

**Table 4:** Changes in secondary outcomes over time.

Clinical Outcomes	Group	T0	T1	T2	T3
**FACIT** (mean ± SD)	Early PR (*N* = 15)	37.9 ± 11.5	40.8 ± 5.2	39.2 ± 9.5	44.4 ± 3.8
	Delayed PR (*N* = 16)	40.3 ± 6.2	40.1 ± 5.8	44.0 ± 4.8	41.9 ± 8.6
**EORTC–function** (mean ± SD)	Early PR (*N* = 15)	80.6 ± 14.1	80.6 ± 10.7	73.2 ± 25.5	85.1 ± 12.6
	Delayed PR (*N* = 16)	79.2 ± 12.2	87.4 ± 7.4	85.5 ± 8.1	81.6 ± 17.5
**EORTC–QoL** (mean ± SD)	Early PR (*N* = 15)	70.6 ± 18.3	59.8 ± 18.2	66.7 ± 18.5	56.9 ± 26.3
	Delayed PR (*N* = 16)	59.9 ± 17.5	59.7 ± 15.0	63.6 ± 12.5	66.7 ± 9.6
**EORTC–symptoms** (mean ± SD)	Early PR (*N* = 15)	19.5 ± 15.8	17.9 ± 12.9	15.6 ± 16.5	11.3 ± 10.2
	Delayed PR (*N* = 16)	17.1 ± 9.1	10.7 ± 7.2	11.2 ± 7.3	15.8 ± 13.9
**6MWT** (mean ± SD)	Early PR (*N* = 15)	456.5 ± 100.2	486.7 ± 60.5	450.6 ± 91.1	Not assessed
	Delayed PR (*N* = 16)	451.4 ± 101.5	492.3 ± 86.3	509.9 ± 93.1	Not assessed
**IPAQ** (mean ± SD)	Early PR (*N* = 15)	665.4 ± 814.7	1527.8 ± 1357.4	2678.2 ± 4173.4	1490.9 ± 1697.8
	Delayed PR (*N* = 16)	1481.2 ± 1832.7	4408.0 ± 4535.9	4533.7 ± 4145.9	2760.1 ± 2400.3

## Data Availability

The data presented in this study are available on request from the corresponding author.
